# Budding yeast relies on G_1_ cyclin specificity to couple cell cycle progression with morphogenetic development

**DOI:** 10.1126/sciadv.abg0007

**Published:** 2021-06-04

**Authors:** Deniz Pirincci Ercan, Florine Chrétien, Probir Chakravarty, Helen R. Flynn, Ambrosius P. Snijders, Frank Uhlmann

**Affiliations:** 1 Chromosome Segregation Laboratory, The Francis Crick Institute, London, UK.; 2 Bioinformatics and Biostatistics Science Technology Platform, The Francis Crick Institute, London, UK.; 3 Proteomics Science Technology Platform, The Francis Crick Institute, London, UK.

## Abstract

Two models have been put forward for cyclin-dependent kinase (Cdk) control of the cell cycle. In the qualitative model, cell cycle events are ordered by distinct substrate specificities of successive cyclin waves. Alternatively, in the quantitative model, the gradual rise of Cdk activity from G_1_ phase to mitosis leads to ordered substrate phosphorylation at sequential thresholds. Here, we study the relative contributions of qualitative and quantitative Cdk control in *Saccharomyces cerevisiae*. All S phase and mitotic cyclins can be replaced by a single mitotic cyclin, albeit at the cost of reduced fitness. A single cyclin can also replace all G_1_ cyclins to support ordered cell cycle progression, fulfilling key predictions of the quantitative model. However, single-cyclin cells fail to polarize or grow buds and thus cannot survive. Our results suggest that budding yeast has become dependent on G_1_ cyclin specificity to couple cell cycle progression to essential morphogenetic events.

## Teaser

A synthetic biology approach reveals why budding yeast needs more than one cyclin to grow and divide.

## INTRODUCTION

The cell division cycle consists of a series of temporally regulated processes that couple cell growth to genome duplication, chromosome segregation, and eventually the birth of two daughter cells. These processes are orchestrated by the oscillatory activity of a master cell cycle regulator, the cyclin-dependent kinase (Cdk) complexes, and their counteracting phosphatases (*1*, *2*). Over the duration of one cell cycle, Cdk complexes phosphorylate hundreds of substrates containing Cdk motifs composed of S/T-P residues with a preferential basic residue K/R at the +3 position. Phosphorylated substrates can, among other fates, become activated or inactivated, change cellular localization, or be targeted for proteasomal degradation. Thus, it is key to successful cell cycle progression that the correct Cdk substrates are phosphorylated at the right time. In addition to phosphorylation, cell cycle regulation is shaped by proteolysis that targets cyclins and other key cell cycle regulators (*3*).

Sequential waves of multiple distinct cyclin-Cdk complexes are typically observed in eukaryotic cells. In the qualitative model for Cdk control of the cell cycle, the substrate specificities of these successively expressed cyclins underpin the ordering of cell cycle events. The budding yeast *Saccharomyces cerevisiae* provides an archetypal example for cyclin waves. A single Cdk catalytic subunit (Cdc28) sequentially forms complexes with three G_1_ phase cyclins (Cln1 to Cln3) followed by two S phase cyclins (Clb5 and Clb6) and lastly four mitotic cyclins (Clb1 to Clb4). These cyclins use hydrophobic interaction surfaces to recognize distinct short linear interaction motifs on their substrates, which promote substrate docking and phosphorylation. Substrate docking motifs are found at a distance from the phosphorylation site and have been described by their amino acid consensus as LP (interacting with Cln1 and Cln2) (*4*–*6*), NLxxxL or K/RxL (Clb5 and Clb6) (*7*–*10*), PxF (Clb3) (*11*), and LxF (Clb2) (*12*) motifs. Docking interactions can also play a role in directing Cdk to a specific subcellular location, e.g., Clb3 localization to the nuclear envelope, spindle poles, and lipid particles (*11*). Despite the regulatory potential of distinct, sequentially expressed cyclins, they display functional overlap and plasticity. For example, early expression of the mitotic cyclin Clb2 can replace the S phase cyclin Clb5, as long as the Cdk inhibitor Swe1 is removed (*13*).

In addition to distinct substrate specificities, cyclins differ in their ability to activate the Cdk kinase. In the order of appearance during cell cycle progression, the budding yeast cyclins Cln2, Clb5, Clb3, and Clb2 confer increasing Cdk activity toward a generic substrate (*4*). This leads to a quantitative increase in overall Cdk activity as cells progress from G_1_ to mitosis and forms the basis for the quantitative model for Cdk control of the cell cycle. This model proposes that cell cycle events are triggered when Cdk activity reaches certain quantitative thresholds, the level needed to trigger S phase being lower than the threshold required for entry into mitosis (*14*). In support of the quantitative model, a single cyclin is sufficient to drive cell proliferation in an engineered fission yeast *S. pombe* strain (*15*). In such a single-cyclin strain, no differential cyclin specificities are available to order cell cycle events. Instead, increasing quantitative Cdk thresholds control S phase and mitosis (*16*, *17*). Whether cell proliferation with a single cyclin is possible in organisms other than fission yeast is not yet known.

In this study, we investigate the relative contributions of qualitative cyclin specificity and quantitative Cdk control to ordering cell cycle progression in budding yeast. We first replace the two S phase and four mitotic cyclins with one mitotic cyclin, Clb2, expressed from an S phase promoter in addition to its endogenous promoter. Ordered S phase and mitosis is maintained in this strain, yet DNA replication is delayed and phosphoproteome analysis reveals a collapse of the intricate phosphorylation landscape that six distinct cyclins normally provide. We then also replace the three G_1_ cyclins with Clb2. Notably, Clb2 alone is able to guide cells from G_1_ through S phase and into mitosis, thus fulfilling key predictions from the quantitative model for Cdk control. However, these single-cyclin budding yeast cells do not polarize or form buds and therefore fail to proliferate. Our results reveal that G_1_ cyclins took on a critical role during the evolutionary diversification of budding yeast cyclins to couple cell cycle progression with essential morphogenetic development.

## RESULTS

### A mitotic cyclin sustains S phase with a delay

**Fig. 1:**
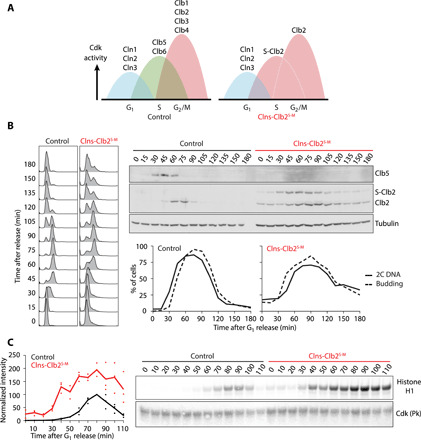
S phase delay in the Clns-Clb2^S-M^ strain. (**A**) Schematic of cyclin waves in wild-type budding yeast and in the Clns-Clb2^S-M^ strain. (**B**) Cell cycle progression of control and Clns-Clb2^S-M^ cells. α-Factor–synchronized cells were followed through one cell cycle before rearrest in the following G_1_. Flow cytometry analysis of DNA content as well as Western blot analysis of Clb5 and Clb2 levels is shown. Clb2 expressed from the *CLB5* promoter was fused to a 6×HA epitope tag, causing its slower migration. Tubulin served as a loading control. The fraction of budded cells over time is shown, as well as the fraction of cells with 2C DNA content. (**C**) Cdk-associated kinase activity against histone H1 was measured following Cdc28 immunoprecipitation by virtue of a Pk epitope tag. A representative autoradiogram and Western blot are shown. The results from three independent experiments are shown; the medians are connected by a line.

Expression of the budding yeast mitotic cyclin Clb2 in place of the S phase cyclin Clb5 supports cell proliferation, even though Cdk phosphorylation of the replication initiation factor Sld2 is compromised (*9*, *13*, *18*, *19*). To better understand the consequences of replacing S phase cyclins with a mitotic cyclin, we established a strain in which two copies of Clb2, expressed under control of the *CLB5* and *CLB2* promoters, were the only source of S phase and mitotic cyclins. The G_1_ cyclins Cln1 to Cln3 remained untouched. We term this the “Clns-Clb2^S-M^” strain (Fig. 1A). We then observed cell cycle progression of the Clns-Clb2^S-M^ strain following synchronization by pheromone α-factor block and release and compared it to a control strain harboring all nine cyclins. Swe1, an inhibitor of mitotic cyclin-Cdk complexes, was removed from both strains to allow unhindered Clb2 activity throughout the cell cycle (*13*).

Following release from the α-factor block, bud formation occurred with similar timing in both the Clns-Clb2^S-M^ and control strains (Fig. 1B). This was expected, as bud formation is controlled by G_1_ cyclins that were present in both strains (*20*–*22*). Clns-Clb2^S-M^ cells expressed Clb2 from the *CLB5* promoter with similar timing to Clb5 expression in control cells. In contrast, Clns-Clb2^S-M^ cells underwent DNA replication 15 min later than the control, as observed by flow cytometry analysis of DNA content (Fig. 1B). This delay occurred despite the fact that Cdk activity, measured against a generic substrate histone H1 in vitro, increased faster and reached higher levels in Clns-Clb2^S-M^ cells (Fig. 1C). The higher Cdk activity level can be explained by the greater potential of Clb2 to activate Cdk, when compared to Clb5 (*4*). These results suggest that early Clb2 expression promotes a faster quantitative increase in Cdk activity but has a lower potential than Clb5 in promoting DNA replication.

Despite the higher Clb2-Cdk activity level, entry into mitosis, as evidenced by metaphase spindle formation, and anaphase onset were also delayed in Clns-Clb2^S-M^ cells (fig. S1A). This is a first indication that both cyclin specificity and quantitative Cdk activity contribute to controlling the budding yeast cell cycle. The specificity of S phase cyclins determines the onset of DNA replication. In their absence, the gradual quantitative increase of Clb2-Cdk activity can also order S phase and mitosis, albeit with altered timings.

In addition to delays in entering S phase and mitosis, Clns-Clb2^S-M^ cells progressed through the cell cycle less synchronously than control cells. Clb2 degradation during exit from mitosis remained sluggish and incomplete. We will return to this observation below.

### Cyclin specificity shapes the Cdk phosphorylation landscape

To survey the impact of cyclin specificity on Cdk substrate phosphorylation, we performed time-resolved phosphoproteome analysis of Clns-Clb2^S-M^ and control strains. Following release from cell synchronization with α-factor, we collected samples at 10-min intervals until cells reached mitosis at 90 min (fig. S1, B and C). Phosphoproteome analysis, using 10 isobaric mass tags, allowed us to follow the abundance changes of 9909 phosphosites. In control cells, 3578 of these sites gained more than 1.5-fold in abundance during progression from G_1_ to mitosis. Among them, 1091 adhered to the minimal S/TP Cdk recognition motif.

**Fig. 2:**
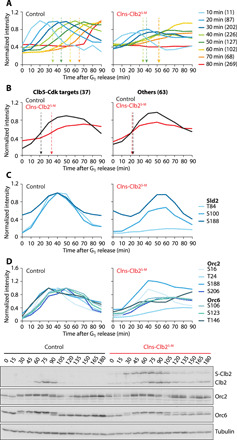
Compromised S phase target phosphorylation in Clns-Clb2^S-M^ cells. (**A**) Median normalized intensity profiles of Cdk targets, grouped by their phosphorylation timing in control cells. Phosphosite number in each category is given in parenthesis. The same groups are shown in the Clns-Clb2^S-M^ strain. Phosphorylation midpoints of the 40 to 70 min categories are indicated by dashed arrows. A complete list of phosphosites can be found in data file S1. (**B**) Median normalized intensity profiles of phosphosites from biochemically identified Clb5-Cdk targets (*9*), as well as sites with similar control phosphorylation timing in targets lacking Clb5 specificity (*9*). Dashed arrows point to phosphorylation midpoints. (**C** and **D**) Normalized intensity profiles of Cdk sites in Sld2, Orc2, and Orc6. Line colors are chosen to approximate those of the time categories in (A). Western blot analyses of Orc2 and Orc6 phosphorylation during synchronous cell cycle progression of wild-type and Clns-Clb2^S-M^ cells are also shown.

We grouped these candidate Cdk phosphosites by the time when they first pass the 1.5-fold enrichment threshold in control cells. This confirmed widely spread-out phosphorylation of Cdk substrates during cell cycle progression (Fig. 2A) (*4*, *17*, *23*–*26*). We then plotted the phosphorylation timing of the same groups of phosphosites in Clns-Clb2^S-M^ cells. While the overall magnitude of phosphorylation changes was smaller, probably because of the inferior cell cycle synchrony of the Clns-Clb2^S-M^ culture, the early phosphorylation waves at 10, 20, and 30 min appeared largely unchanged. This was expected from the presence of unaltered G_1_ cyclins in both strains. Between 40 and 70 min, the time window between S phase and entry into mitosis, a large number of sites were sequentially phosphorylated in the control. In Clns-Clb2^S-M^ cells, the time resolution between these phosphorylation waves collapsed and was replaced by a slow gradual increase of the sites over time. This illustrates how distinct S phase and mitotic cyclins enable the execution of a time-resolved cell cycle phosphorylation program.

Phosphorylation events occurred in sequential waves in control cells, reflecting consecutive waves of cyclin expression (Figs. 1B and 2A). In contrast, sites that were phosphorylated from S phase onward remained phosphorylated for prolonged periods in Clns-Clb2^S-M^ cells, again mirroring Clb2 levels that persisted starting from S phase onward until mitosis. This suggests that not only sequential cyclin synthesis but also sequential cyclin proteolysis contributes to shaping the cell cycle phosphorylation landscape.

Last, we observed that the last, mitotic phosphorylation wave at 80 min was greatly subdued in Clns-Clb2^S-M^ cells. This is unexpected when considering that mitotic Clb2-Cdk activity is greater in Clns-Clb2^S-M^ cells compared to the control. Possible explanations include that mitotic cyclins in addition to Clb2 are important to achieve mitotic Cdk phosphorylation or that the regulation of Cdk counteracting phosphatases is affected by the absence of S phase cyclins. In addition, delayed cell biological events in Clns-Clb2^S-M^ cells might delay downstream Cdk substrate phosphorylation. Together, these observations manifest the importance of a full cyclin complement in diversifying the budding yeast cell cycle phosphorylation program. A hierarchical clustering analysis of phosphosite behavior in control and Clns-Clb2^S-M^ cells confirmed the above conclusions and revealed further details of phosphosite behavior (fig. S2).

### Impact of cyclin specificity on S phase targets

We next analyzed the consequences of S phase cyclin loss on the in vivo phosphorylation of a set of in vitro determined Clb5-Cdk substrates (*9*). Our control dataset contained 37 phosphosites on 13 of the 14 described substrates. These sites displayed early phosphorylation at approximately 20 min, which was delayed by more than 10 min in Clns-Clb2^S-M^ cells (Fig. 2B). In contrast, 63 phosphosites on proteins that were phosphorylated with similar early timing in our control strain, but showed no in vitro Clb5 preference (*9*), retained unaltered phosphorylation timing.

Of the in vitro Clb5-Cdk targets, Sld2 is a key replication initiation factor whose timely in vivo phosphorylation has previously been reported to depend on Clb5 (*9*, *13*, *18*, *19*). The single phosphosite resolution of our dataset revealed that the T84 site that is critical for replication initiation (*27*) was hardly phosphorylated in Clns-Clb2^S-M^ cells (Fig. 2C). In contrast, other Cdk phosphorylation sites on Sld2 were less affected.

Orc2 and Orc6, whose Cdk phosphorylation is part of the mechanism that blocks re-replication (*28*, *29*), are also in vitro Clb5-Cdk targets (*9*). Our phosphoproteome analysis contained numerous Cdk sites on both Orc subunits. Their phosphorylation in early S phase was affected to various extents by the absence of Clb5 (Fig. 2D). Among the most affected residues were Orc2 T24 and S206, which are located in predicted disordered regions of the protein, 10 and 19 amino acids upstream of RxL and KxL motifs, respectively. A small delay and reduction of Orc2 and Orc6 phosphorylation in the absence of Clb5 was also seen by Western blotting. Together, these results demonstrate the importance of S phase cyclin specificity in regulating important DNA replication factors. They further illustrate how phosphosites can be differentiated within Cdk substrates by local cyclin docking motifs.

### Impact of cyclin specificity on G_2_ and mitotic targets

**Fig. 3:**
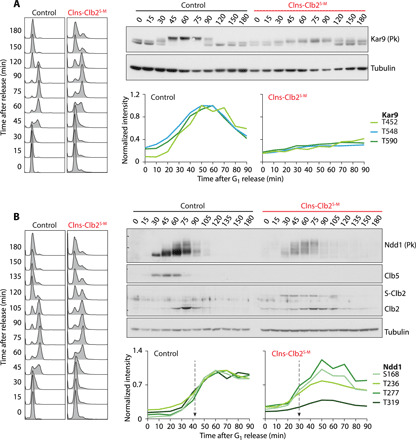
Cyclin specificity shapes the Cdk phosphorylation landscape. (**A**) Contribution of Clb3-specific substrate interactions. Kar9 phosphorylation was assessed during synchronous cell cycle progression in control and Clns-Clb2^S-M^ cells by Western blotting. Normalized intensity profiles from the phosphoproteome data of the indicated sites are also shown. (**B**) as in (A) but the effect of early Clb2 expression on Ndd1 phosphorylation was evaluated. The dashed arrows point to the phosphorylation midpoints.

Clns-Clb2^S-M^ cells lacked not only S phase cyclins but also the mitotic cyclins Clb1, Clb3, and Clb4. The spindle positioning factor Kar9 is thought to be a Clb3 and Clb4 target (*30*). Its G_2_-specific phosphorylation was greatly impaired in Clns-Clb2^S-M^ cells, both when judged by its electrophoretic mobility shift and the phosphoproteome data (Fig. 3A). We also surveyed Cdk phosphosites that lie in the vicinity of predicted PxF Clb3 docking motifs in the cell polarity factor Boi1 and the nuclear envelope and spindle pole protein Csa1 (*11*). These phosphosites were again greatly affected in the Clns-Clb2^S-M^ strain (fig. S3A).

Last, we investigated the consequence of earlier than usual Clb2 expression in the Clns-Clb2^S-M^ strain. The transcription factor Ndd1 is an important Clb2 target that contributes to Clb2 transcriptional autoregulation (*31*, *32*). Ndd1 phosphorylation was advanced in Clns-Clb2^S-M^ cells, both when judged by its electrophoretic mobility shift as well as the phosphoproteome data (Fig. 3B). Additional Cdk targets containing predicted Clb2 docking motifs (*12*), the replication initiator Cdc6 and the formin Bni1, again displayed advanced phosphorylation of sites close to their LxF sequences (fig. S3B). Together, these analyses reveal the extent by which cyclin specificity, via docking site interactions, shapes the multifaceted Cdk phosphorylation landscape during cell cycle progression.

### A DNA damage signature and reduced fitness of Clns-Clb2^S-M^ cells

**Fig. 4:**
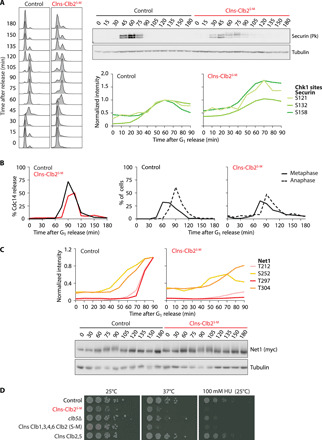
Signs of a DNA damage response in Clns-Clb2^S-M^ cells. (**A**) Delayed securin degradation following its phosphorylation on Chk1 kinase sites. Securin levels in synchronized cultures of control and Clns-Clb2^S-M^ cells were analyzed by Western blotting. Normalized intensity profiles of three Chk1 kinase target sites contained in the phosphoproteome data are shown. (**B**) Delayed Cdc14 release in Clns-Clb2^S-M^ cells. Cdc14 fused to a Pk epitope tag was visualized by indirect immunofluorescence during a time course experiment as in (A). One hundred cells were scored for loss of discernible nucleolar Cdc14 accumulation at each time point, as well as for the presence of short metaphase or long anaphase spindles. (**C**) Compromised Net1 phosphorylation. The Net1 phosphorylation status was analyzed during synchronous cell cycle progression of control and Clns-Clb2^S-M^ cells by Western blotting. Normalized intensity profiles of four Cdk phosphorylation sites that regulate Cdc14 release are shown. (**D**) Reduced fitness of Clns-Clb2^S-M^ cells. Tenfold serial dilutions of cells with the indicated genotypes were spotted on YPD plates, with or without added hydroxyurea (HU), and grown for 3 days at the indicated temperatures.

Clb2 degradation appeared sluggish, indicative of compromised mitotic exit in Clns-Clb2^S-M^ cells (Fig. 1B). This could be an indication of cell cycle checkpoint activation. Examination of securin levels, which acts as downstream effector of budding yeast cell cycle checkpoints, revealed markedly delayed securin degradation in Clns-Clb2^S-M^ cells (Fig. 4A). Furthermore, securin showed noticeably increased phosphorylation at target sites of the Chk1 checkpoint kinase, which are known to stabilize securin (Fig. 4A) (*33*). These observations open the possibility that a DNA damage signal in Clns-Clb2^S-M^ cells delays mitotic exit. On the contrary, we did not find evidence for activation of two other budding yeast cell cycle checkpoints, the S phase and mitotic checkpoints (fig. S4, A and B) (*34*, *35*).

Persisting securin impedes anaphase onset and mitotic exit by preventing separase from cleaving cohesin and from activating the Cdc14 phosphatase (*36*, *37*). Consistently, we found that anaphase onset and Cdc14 release from its inhibitory sequestration in the nucleolus was delayed by around 15 min in Clns-Clb2^S-M^ cells (Fig. 4B). Separase facilitates Cdc14 release by promoting Cdk phosphorylation of the nucleolar Cdc14 inhibitor Net1 (*37*, *38*). Accordingly, we found that Net1 phosphorylation was reduced and delayed, both when looking at the Net1 electrophoretic mobility shift and the phosphoproteome data of four of six Net1 Cdk phosphosites that have been implicated in Cdc14 release (Fig. 4C) (*38*).

A DNA damage signal is an indication of endogenous stress that might render cells sensitive to additional exogenous challenges. We found that Clns-Clb2^S-M^ cells showed marked growth defects at a higher temperature or in the presence of the DNA replication inhibitor hydroxyurea (Fig. 4D). This growth analysis also revealed that much of the sensitivity arose from replacing the S phase cyclin Clb5 with Clb2, rather than from the absence of Clb1, Clb3, and Clb4. Together, these results demonstrate the importance of cyclin specificity, and especially the presence of both S phase and mitotic cyclins, for faithful and timely cell cycle progression and cell fitness in budding yeast.

### Cell cycle progression with a single cyclin

We next asked whether budding yeast cells can proliferate with Clb2 as the sole source of cyclin-Cdk activity. Loss of budding yeast G_1_ cyclins is lethal, but growth is thought to be restored by simultaneous removal of the stoichiometric Cdk inhibitor Sic1 (*39*). We successfully eliminated the G_1_ cyclins Cln1 and Cln3, as well as Sic1, from Clns-Clb2^S-M^ cells, thereby creating a Cln2-Clb2^S-M^ strain (fig. S5A). However, attempts to additionally remove Cln2 failed, in line with another recent report that cells lacking G_1_ cyclins and Sic1 are unviable (*40*). Cln2-Clb2^S-M^ cells showed markedly delayed Cln2 expression and DNA replication, compared to the corresponding *swe1*∆ *sic1*∆ control (fig. S5B), consistent with the known role of G_1_ cyclins to promote each other’s expression (*41*). We now added a third copy of Clb2, expressed under control of the *CLN2* promoter, to create a Cln2-Clb2^G1-S-M^ strain. This resulted in early Clb2 accumulation that coincided with Cln2. The early presence of Clb2 advanced Cln2 expression, compared to Cln2-Clb2^S-M^ cells. It also advanced DNA replication (fig. S5B). It was previously thought that Clb2 represses G_1_ cyclin synthesis, at least at later cell cycle stages when Clb2 reaches higher levels (*42*). Following its early expression from the *CLN2* promoter, it appears that Clb2 promoted G_1_ cell cycle progression.

**Fig. 5:**
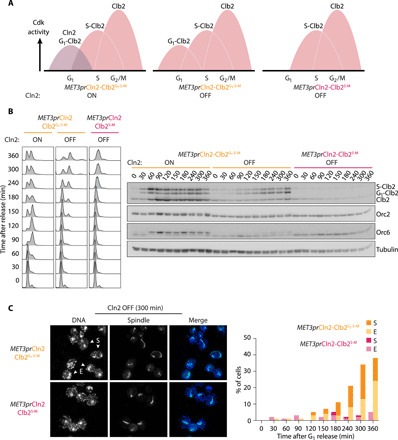
Cell cycle progression with a single cyclin. (**A**) Schematic of cyclin waves in the *MET3pr*Cln2-Clb2^G1-S-M^ strain with active or repressed *MET3* promoter, as well as in the repressed *MET3pr*Cln2-Clb2^S-M^ strain. (**B**) Cell cycle characteristics with a single cyclin. α-Factor synchronized cells of the indicated genotypes were released into medium lacking (Cln2 ON) or containing methionine (Cln2 OFF). Flow cytometry analysis of DNA content is shown together with Western blots of cyclin expression and cell cycle markers. Clb2 expressed from the *CLN2* promoter was fused to a 3×HA epitope tag, leading to migration between CLB5 promoter expressed 6×HA epitope–tagged Clb2 and endogenous untagged Clb2. Tubulin served as a loading control. (**C**) Mitosis inside single-cell bodies in the single-cyclin strain. Fields of *MET3pr*Cln2-Clb2^G1-S-M^ and *MET3pr*Cln2-Clb2^S-M^ cells with Cln2 OFF from the 300 min time point, stained for the mitotic spindle and DNA. One hundred cells at each time point were scored for elongated (E) or segregated (S) nuclei.

We next studied whether G_1_-expressed Clb2 could replace Cln2. To do so, we placed a methionine-repressible *MET3* promoter in front of the *CLN2* gene to create a *MET3pr*Cln2-Clb2^G1-S-M^ strain (Fig. 5A). When released from α-factor synchronization into methionine-free medium, Cln2 expression was maintained, resulting in cyclin accumulation followed by S phase. When Cln2 expression was repressed by release into medium supplemented with methionine, cells progressed more slowly through G_1_ (Fig. 5B). Nevertheless, Clb2 accumulated, leading to Cdk substrate phosphorylation and eventually DNA replication. This suggests that Clb2 can replace Cln2 and promote progression through G_1_ and into S phase, albeit with a delay. Cell cycle progression was driven by Clb2, rather than residual Cln2 that might have persisted following *MET3* promoter shutoff, as cells without *CLN2* promoter-expressed Clb2 remained stably blocked in G_1_ and showed neither cyclin expression nor Cdk substrate phosphorylation.

### Single-cyclin cells complete a nuclear division cycle

We next observed how cells expressing only Clb2 progressed from S phase to mitosis. To this end, we performed immunofluorescence imaging of mitotic spindles, together with nuclei, at time points following DNA replication. This revealed the formation of bipolar spindles in almost half of the cells, which elongated and segregated DNA into two equal masses. However, this process of apparent chromosome segregation took place in large, single-cell bodies (Fig. 5C). We did not observe bud formation and consequently no cell division could take place. These observations suggest that Clb2 as the sole cyclin can instruct genome duplication and segregation but fails in producing progeny by cell division. Flow cytometry analysis of DNA content revealed that, at later time points, a fraction of cells underwent another round of genome doubling, suggesting that they had completed progression through one biochemical cell cycle and entered the next. However, because of their failure to generate buds and divide, *MET3pr*Cln2-Clb2^G1-S-M^ cells were not able to proliferate on methionine-containing medium where Clb2 provides the only source of Cdk activity (fig. S5C).

### The role of Cln2 in cell proliferation

**Fig. 6:**
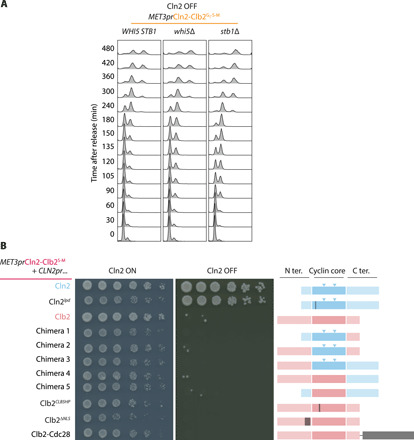
Dissecting the essential nature of G_1_ cyclins. (**A**) Transcriptional inhibitors delay G_1_ progression. Cells of the indicated genotypes were synchronized by α-factor treatment and released. Flow cytometry analysis of DNA content was used to monitor G_1_ progression and entry into S phase. (**B**) Analysis of G_1_ cyclin features. Fourfold serial dilutions of cells with indicated genotypes were spotted onto CSM without methionine (Cln2 ON) or YPD (Cln2 OFF) plates and grown for 3 days at 25°C. Schematics represent the cyclin variants expressed under control of the *CLN2* promoter. Cln2 (blue) and Clb2 (red) are divided into their N-terminal, cyclin core, and C-terminal parts. Two Cln2-specific loop insertions are highlighted by arrowheads. Locations of engineered gene alterations are highlighted in dark gray.

To gain insight into why Clb2 could not fully replace Cln2 in promoting cell proliferation, we considered differences between the two cyclins. An important role of G_1_ cyclins is activation of the G_1_ transcriptional program, which in budding yeast involves phosphorylation and thereby inactivation of the transcriptional repressors Whi5 and Stb1 (*43*–*45*). To overcome transcriptional inhibition, we deleted the genes encoding Whi5 or Stb1 in the *MET3pr*Cln2-Clb2^G1-S-M^ background. This did not noticeably change the timing of G_1_ progression when Cln2 was active (fig. S6A). Notably, when Cln2 was repressed, G_1_ progression and S phase entry markedly accelerated in the absence of Whi5 or Stb1 (Fig. 6A). Despite the successful up-regulation and acceleration of cyclin gene transcription, cells remained unsuccessful at forming buds and were unable to proliferate on methionine containing medium. Deletion of the MluI cell cycle box binding factor (MBF) transcription factor subunit Mbp1, which allows for promiscuous expression of its G_1_ targets (*46*), also did not restore viability (fig. S6B).

In addition to functional distinctions between Cln2 and Clb2, we considered structural differences. While cytoplasmic Cln2 is important for efficient budding (*47*), Clb2 is enriched in the nucleus. To increase cytoplasmic Clb2 levels, we deleted its nuclear localization signal (*48*), but this was insufficient to restore viability without Cln2 (Fig. 6B and fig. S6C). We also covalently fused Clb2 to the Cdc28 kinase subunit, an approach that facilitated constitutive cyclin-Cdk complex formation in fission yeast (*16*), but that also did not reinstate viability.

Overexpression of the S phase cyclin Clb5 has been reported to compensate for the absence of G_1_ cyclins (*49*, *50*). The overall similar architecture of Clb5 and Clb2 made it possible to replace Clb2’s hydrophobic substrate binding pocket with that of Clb5. However, the resultant Clb2*^CLB5HP^* was unable to promote cell proliferation without Cln2 (Fig. 6B and fig. S6C).

To address the importance of Cln2-specific substrate targeting in an alternative way, we made use of an LP motif docking site mutation in Cln2, Cln2*^lpd^*, that reduces Cln2-specific substrate interactions and phosphorylation (*6*). We then analyzed whether Cln2*^lpd^* was able to sustain cell growth following wild-type Cln2 depletion in *MET3pr*Cln2-Clb2^S-M^ cells. Unexpectedly, Cln2*^lpd^* supported cell proliferation to a similar extent as wild-type Cln2. Therefore, the features of Cln2 that distinguish it from Clb2 in promoting budding and cell proliferation must lie outside its LP motif docking site.

In an attempt to narrow down the region of Cln2 that is required to promote budding and sustain cell proliferation, we created five Cln2-Clb2 chimeras on the basis of a structure-based alignment of Cln2 and Clb2. This involved swaps of the cyclin core, as well as the N- and C-terminal extensions. While the resultant chimeras were expressed as stable proteins under control of the *CLN2* promoter (Fig. 6B and fig. S6C), none was able to support cell growth following Cln2 repression. The molecular features that make Cln2 essential therefore remain to be further explored.

### Cln2 specificity promotes cell polarization and budding target phosphorylation

**Fig. 7:**
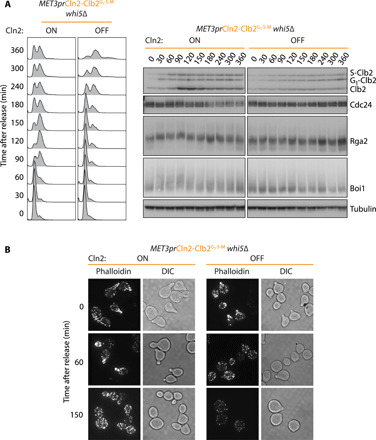
Cln2 is required for cell polarization and budding pathway target phosphorylation. (**A**) Cln2 promotes budding pathway target phosphorylation. Electrophoretic mobility shifts, indicative of phosphorylation, of the denoted proteins were assessed during synchronous cell cycle progression of *MET3pr*Cln2-Clb2^G1-S-M^ cells in CSM medium lacking (Cln2 ON) or containing (Cln2 OFF) methionine. Tubulin served as a loading control. (**B**) Cln2 is required for cell polarization. Samples from (A) were processed for rhodamine-phalloidin staining at the indicated time points. Fluorescence is shown next to differential interference contrast (DIC) images.

Given the failure of cells expressing only Clb2 to grow buds, we wondered whether Cln2 was specifically required for phosphorylation of budding pathway factors. Three Cdk targets associated with the Cdc42 guanosine triphosphatase that is central to cell polarization, Boi1, Rga2, and Cdc24, are more efficiently targeted by Cln2-Cdk in vitro than by Clb2-Cdk (*51*). We synchronized *MET3pr*Cln2-Clb2^G1-S-M^ *whi5*∆ cells by α-factor treatment and released them into medium that allowed or suppressed Cln2 expression. As soon as cyclins accumulated in Cln2-expressing cells, Boi1, Rga2, and Cdc24 showed electrophoretic mobility shifts characteristic of their phosphorylation (Fig. 7A) (*51*). On the other hand, no mobility shift was detected in cells lacking Cln2, even at later times when Clb2 levels increased, and cells entered S phase. This suggests that Cln2 specifically phosphorylates positive regulators of bud formation.

To directly visualize cell polarization, we stained cells with phalloidin to label actin filaments. α-Factor–treated cells displayed actin accumulation at their polarized shmoo tips. Following release from the arrest, Cln2-expressing cells retained polarity at the sites of bud formation (Fig. 7B). In contrast, polarization was lost from the shmoo tips in cells lacking Cln2. Instead, actin was found in patches dispersed around the cell circumference. Polarization was not visibly regained in these cells at any time during the experiment. Together, these results suggest that Cln2 plays a unique role during bud formation because it phosphorylates targets in the cell polarity and budding pathways that cannot be reached by Clb2-Cdk complexes.

## DISCUSSION

Here, we analyze the relative contributions of cyclin specificity and quantitative Cdk control to budding yeast cell cycle progression. We find evidence for important contributions of both mechanisms. On the one hand, the quantitative increase of a single mitotic cyclin can replace all other cyclins and bring about ordered progression from G_1_ through S phase and into mitosis. Therefore, key cell cycle targets must have been phosphorylated in the correct order by a single source of Cdk activity. Possible mechanisms that explain this ordering include phosphatase thresholds or a multisite phosphorylation code (*2*, *52*, *53*). On the other hand, many aspects of the multifaceted Cdk phosphorylation landscape depend on individual cyclins and their ability to recognize distinct small linear substrate interaction motifs. Losing this added level of regulation results in a substantial fitness reduction when considering cell cycle control of S phase and mitosis. Our time-resolved phosphoproteome analysis expands on previously published datasets (*26*, *54*) and provides a comprehensive overview of how cyclin specificity contributes to cell cycle phosphorylation dynamics.

When we also replace G_1_ cyclins by the single mitotic cyclin, cells can complete a nuclear division cycle but fail to form viable progeny. The reason for the inability of a single mitotic cyclin to support cell proliferation was its failure to promote cell polarization and budding. G_1_ cyclins use an LP motif docking mechanism that is known to contribute to cell polarization (*6*). However, the Cln2 LP docking site was not required to convey its critical role. Could it therefore be that not the absence of Cln2 but premature Clb2 expression interfered with cell polarization in our single-cyclin experiment? Clb2 promotes depolarization of the cortical actin network, at least at the time when cells have reached G_2_ (*20*). Clb2 has furthermore been suggested to prevent bud formation by repressing G_1_ gene expression (*55*). However, we consider an inhibitory role of Clb2 in G_1_ unlikely. In our experiments, Clb2 did not prevent bud formation as long as Cln2 was also present. On the contrary, G_1_-expressed Clb2 replaced other missing G_1_ cyclins, accelerating cyclin expression and S phase onset. Within the context of G_1_ cells, Clb2 therefore contributes positively to most aspects of G_1_ progression, except for cell polarization and budding. On the basis of this evidence, we suggest that Cln2 carries a critical role in promoting phosphorylation of cell polarization and budding pathway targets that Clb2 cannot reach. Cln2 appears to do so using substrate interactions that go beyond those provided by the LP docking site. Further studies will explore the nature of these interactions and whether they are shared with other G_1_ cyclins (*56*).

Will it be possible to engineer a single cyclin that can successfully drive the whole of the budding yeast cell division cycle? While Cln2 is uniquely able to drive cell polarization and budding, it is a poor overall Cdc28 kinase activator (*4*). We do not yet understand what differentiates cyclins when it comes to quantitative Cdk activation and whether or not Cln2’s unique substrate specificity necessitates weaker Cdk kinase activation. However, because of its limited kinase activation potential, Cln2 alone is unlikely sufficient to drive later cell cycle events. In contrast, instances have been reported when later expressed cyclins can take over G_1_ cyclin function (*39*, *49*, *50*). Notably, a stabilized version of Clb3 can compensate for the absence of both G_1_ and S phase cyclins (*40*). At first sight, Clb3 appears unable to replace Clb1 and Clb2. However, Clb3 shows the least nuclear concentration among the budding yeast cyclins (*53*) and localization is often important for cyclin function (*57*, *58*). Therefore, in future studies, it will be interesting to test whether increasing nuclear accumulation of Clb3 might allow establishment of a single-cyclin budding yeast strain. Such a tool holds promise to reveal more about how cyclin specificity and quantitative Cdk regulation coexist.

Cyclins as cell cycle regulators likely emerged after the divergence of eukaryotes from archaea and bacteria (*59*). From there, G_1_ cyclins have diverged faster than S phase and mitotic cyclins (*60*). It appears that, during their evolution, G_1_ cyclins have acquired specialized functions by recognizing substrate docking motifs different from S phase and mitotic cyclins. LP motif docking is a conserved feature among fungal G_1_ cyclins (*61*), consistent with the notion that ancestral fungi already made use of at least two different cyclin families (*62*). In the future, it will be interesting to understand what triggered the need for harboring more than one cyclin and whether the development of morphogenetic changes such as bud formation created such a need. Fission yeast cells that can live with only one cyclin display a simple morphogenetic life cycle of cell elongation and fission (*15*, *16*). Last, it would be interesting to know when G_1_ cyclins diverged from mitotic cyclins in relation to the separation of the fungi and animal kingdoms. While the mammalian cell cycle is able to progress without input from G_1_ (D-type) cyclins (*63*, *64*), individual D-type cyclins have taken on crucial organ-specific functions (*65*, *66*). It could be that G_1_ cyclins evolved to link cell cycle progression to cell biological events that require G_1_-specific input from the cell cycle control machinery, which cannot be provided by quantitative Cdk control.

## MATERIALS AND METHODS

### Yeast strains and culture

Budding yeast strains were of the W303 background and are listed in table S1. Gene deletions were performed using either polymerase chain reaction (PCR)–based gene targeting (*67*) or CRISPR-Cas9–based genome editing (*68*). For the latter, gene-specific guide RNAs (gRNAs) were designed and cloned into the pML104 vector as described (*68*). The vector was then cotransformed with a double-stranded DNA fragment consisting of successive 150 base pairs upstream and downstream of the targeted gene. Positive transformants were selected on medium lacking uracil and then counter selected on medium containing 5-fluoroorotic acid to allow recycling of the CRISPR-Cas9–gRNA plasmid for further rounds of editing. Epitope tagging of endogenous gene loci and promoter substitutions were performed using PCR-based methods (*69*). Details of all the additional DNA constructs used for constructing cyclin variants can be found in table S1. Cells were grown in rich YP (yeast extract and peptone) medium supplemented with 2% glucose (YPD) or complete supplement mixture (CSM) medium lacking methionine supplemented with 2% glucose, both at 25°C. Mating pheromone α-factor was used for cell synchronization in G_1_ as described (*70*). After collecting an aliquot from the arrested culture (time point 0), cells were released by filtration and resuspended in fresh medium, and further samples were collected at regular intervals. In case of a rearrest in the subsequent G_1_, cells were treated with α-factor (7 μg/ml) after initiation of budding and every following hour. Each cell cycle experiment presented here was repeated on at least two and typically more independent occasions, and a representative experiment is shown.

### Western blotting

Protein extracts for Western blotting were prepared following cell fixation with trichloroacetic acid and bead beating. Extracts were then separated by SDS–polyacrylamide gel electrophoresis (PAGE) and transferred to nitrocellulose membranes. Antibodies used for Western detection were α-Clb5 (Santa Cruz Biotechnology, sc20170), α-Clb2 (Santa Cruz Biotechnology, sc9071), α-Sic1 (Santa Cruz Biotechnology, sc50441), α-Orc6 (clone SB49), α-Orc2 [a gift from S. P. Bell (*71*)], α-Rga2 and α-Boi1 [a gift from D. McCusker (*51*)], α-Cdc24 [a gift from M. Peter (*72*)], α-myc (clone 9E10), α–hemagglutinin (HA) (clone 12CA5), α-Pk (Bio-Rad, clone SV5-Pk1; Abcam, ab15828), and α-tubulin (Crick cell services, clone TAT-1).

### Immunofluorescence microscopy

Indirect immunofluorescence was performed on formaldehyde fixed cells as described (*24*). For mitotic spindle staining, an α-tubulin (Abcam, clone YOL 1/34) antibody was used. Cdc14, fused to a Pk epitope, was detected with an α-Pk antibody (Bio-Rad, clone SV5-Pk1). F-acting detection was performed using formaldehyde fixed cells, washed with phosphate-buffered saline and stained with 0.66 μM rhodamine–conjugated phalloidine (Thermo Fisher Scientific). Cells were washed again and resuspended in a drop of mounting medium containing 4′,6-diamidino-2-phenylindole. Fluorescent images were acquired as serial sections along the *z* axis using a DeltaVision imaging system (Applied Precision) on the basis of an Olympus IX-71 microscope. Image stacks were processed using the quick projection function in SoftWoRx.

### Flow cytometry analysis of DNA content

Cells were fixed in 70% ethanol overnight at 4°C, treated with ribonuclease (RNase) (0.1 mg/ml) in 50 mM tris-HCl (pH 7.5) overnight at 37°C, then stained with propidium iodide, and sonicated before analysis on a LSRFortessa cell sorter (BD Biosciences). Ten thousand cells per sample were counted and analyzed using FlowJo.

### In vitro kinase assay

Cells harboring Pk epitope–tagged Cdc28 were grown and synchronized as described above. Samples were collected every 10 min by centrifugation and snap-frozen in liquid nitrogen. Proteins were extracted by bead beating in lysis buffer [50 mM Hepes/KOH (pH 7.5), 150 mM NaCl, 0.2% Triton X-100, 2.5 mM MgCl_2_, 10% glycerol, 0.5 mM tris(2-carboxyethyl) phosphine (TCEP), 4-(2-aminoethyl) benzenesulfonyl fluoride (AEBSF) (120 μg/ml), including benzonase, RNase A, and a cOmplete (Roche) protease inhibitor tablet]. Extracts were cleared by centrifugation, and 800 μg of protein extract per sample was incubated with 1 μg of α-Pk (Bio-Rad, clone SV5-Pk1) antibody for 30 min on a rotating wheel at 4°C. Protein A Dynabeads (20 μl) per sample were then added and incubated for further 30 min. Beads were extensively washed in lysis buffer and equilibrated in kinase buffer [50 mM Hepes/KOH (pH 7.5), 150 mM NaCl, 10 mM MgCl_2_, 0.05% Triton X-100, and bovine serum albumin (0.25 mg/ml)]. Histone H1 phosphorylation reactions were then carried out in kinase buffer containing histone H1 (0.33 mg/ml; Sigma-Aldrich) and 0.66 mM adenosine 5′-triphosphate (ATP), including 11 nM γ^33^P-ATP (Hartmann Analytics) for 15 min at 30°C. Reactions were terminated by addition of SDS-PAGE loading buffer and boiling at 95°C for 5 min. Proteins were resolved on 4 to 15% Criterion TGX precast midi gradient gels (Bio-Rad) and transferred to a nitrocellulose membrane. Membranes were exposed to a Phosphorimager screen (GE Healthcare), and phosphorylation of histone H1 was quantified using ImageQuant. To control for Cdc28 immunoprecipitation efficiency, the membranes were then processed for Western blotting using the α-Pk (Abcam, ab15828) antibody. The H1 phosphorylation signal was normalized to Cdc28 levels, before comparing all values to the 80-min time point in the control strain, when Cdk showed its maximum activity in the control.

### Tandem mass tag proteomics sample preparation

Cells were grown and synchronized as described above. Samples were taken in 10-min intervals from 0 to 90 min and fixed in trichloroacetic acid. Cells were washed with acetone, resuspended in lysis buffer [50 mM ammonium bicarbonate, 5 mM EDTA (pH 7.5), and 8 M urea], and broken by glass bead beating. Extracts were cleared by centrifugation. Protein extract (200 μg) per sample was processed for mass tag labeling and mass spectrometry as described (*73*). Alternating pairs of control and Clns-Clb2^S-M^ samples were placed into two groups for labeling with TMT10plex reagents (Thermo Fisher Scientific). Compare extended data (fig. S1B) for further details.

### Phosphoproteomics data analysis

The phosphoproteome data were filtered to retain phosphorylated peptides containing a single phosphorylated site and a localization probability score greater than 0.75. Most of the phosphosites were detected in both TMT10plex groups that covered alternating time points, thus covering all 10 time points. Phosphosites that were detected in only one of the two TMT10plex groups were also included in the analysis after the missing phosphosite intensities were inferred by imputation. Missing values were replaced by the mean of the two adjacent time points. Smoothing was then performed by replacing each technical or imputed value by the mean of the two adjacent time points. For both imputation and smoothing of the 0- and 90-min time points, only the 10- and 80-min values, respectively, were used. All phosphosite intensities were then transformed to a linear scale.

For the assignment of Cdk target sites that become phosphorylated between G_1_ and mitosis, the first and last time point of the experiment, the following rules were applied to the control sample. For each site, phosphosite intensities were normalized to the lowest phosphosite intensity. Those phosphosites whose intensity increased by more than 1.5-fold over two consecutive time points, following the time point with the lowest intensity, were categorized as phosphorylated. In addition, if sites showed their minimal intensity at 30 min or later, then we returned to time point 0 and asked whether the intensity increased more than 1.5-fold over time point 0 in two consecutive time points before reaching the minimum. These sites were included on the basis of their early phosphorylation. Any sites with a higher intensity at time point 0 compared to 90 min were eliminated. Sites were then filtered to adhere to the minimal S/TP Cdk consensus motif.

To ascribe phosphorylation timings to Cdk sites, we first identified the time point with the highest intensity following the time point with the lowest intensity (or between the value at time point 0 and the maximum). Half of the difference between these two values was considered the phosphorylation threshold. The phosphorylation time is then the first time that the intensity passes the threshold.

After these analyses were complete, we then normalize all control phosphosite intensities between the lowest and highest intensity, ranging from 0 to 1. Phosphosite intensities in the Clns-Clb2^S-M^ strain are in all cases relative to the control. If the average of more than one phosphosite is depicted for a phosphosite category, then we plot the median.

To assign phosphorylation midpoints to groups of phosphosites, we plot the median values at each time. We then determine the minimum and maximum and consider half of the difference as the phosphorylation threshold. The time at which the threshold is passed is then given by a simple geometric fit between the two intensities and time points before and after the threshold is passed.

Heatmaps were generated using phosphosites grouped by their phosphorylation timing in the control. Intensity values per phosphosite represented by rows were clustered using a Euclidean distance matrix and McKuitty clustering, on the basis of phosphosite behavior in the Clns-Clb2^S-M^ strain. Rows were divided into three groups using K means. Heatmaps were generated using the ComplexHeatmap package in R (version 3.6.0).

The search for cyclin docking motifs in predicted disordered protein regions was carried out using the SlimSearch4 tool (http://slim.icr.ac.uk/slimsearch/) (*74*) and a IUPRED disorder score > 0.3.

## SUPPLEMENTARY MATERIALS

Supplementary material for this article is available at http://advances.sciencemag.org/cgi/content/full/7/23/eabg0007/DC1

## Appendix

View/request a protocol for this paper from *Bio-protocol*.
